# Trends in asthma mortality in Brazil in the 0-4 and 5-34-year age groups

**DOI:** 10.1186/1939-4551-8-S1-A48

**Published:** 2015-04-08

**Authors:** Gustavo Graudenz, Dominique Carneiro

**Affiliations:** 1 Nove De Julho University, Brazil

## Background

Bronchial asthma is a chronic disease with large prevalence rates in Brazil and most industrialized countries. Asthma deaths are rare but, in most cases, preventable with appropriate early diagnosis and disease control. The objective of this study was to update trends in asthma mortality in Brazil according to age groups of 0 to 4 and 5 to 34 years.

## Methods

Data on asthma mortality were obtained from the Mortality Information System - SIM/DATASUS (*Departamento de Informática do SUS* – Brazilian Health System Database), using the International Classification of Diseases codes J45 and J46 for the period from 1980 to 2010. An ecological time-series study was conducted to analyze time trends in standardized mortality rates of asthma, using regression models for the 0-4 and 5-34-year age groups.

## Results

The asthma deaths rates in the age group 0 to 4 years showed a decrease of 78.8% and dropped from 26.1 to 4.0% of all deaths group due to asthma. Asthma rates in 5 to 34 year age group also recorded declines of 75.3%, ranging from 8.0 to 12.2% of the total of asthma deaths. There was a linear, decreasing trend in asthma mortality from 1980 to 2010 in Brazil in both age groups, whereas in the general population there was a third order polynomial trend. Discussion: Asthma mortality in the population ranging from 0 to 34 years of age showed a linear and constant decrease, but the rate of decrease was greater in the 0-4-year age group. The group aged 5 to 34 years also showed a linear decline in mortality, but slower than the previous group. The linear decrease found in both age groups contrasts with the heterogeneous asthma mortality trend in the general population in Brazil. The causes of this asthma-related mortality decline in younger age groups are still matter of debate.

## Conclusions

This study showed a continuous decrease in asthma mortality in Brazil in the 0-34-year age groups.

